# Pulsed Electromagnetic Field Therapy Alters the Genomic Profile of Bladder Cancer Cell Line HT-1197

**DOI:** 10.3390/jpm15040143

**Published:** 2025-04-04

**Authors:** Maxwell Sandberg, Wyatt Whitman, Randall Bissette, Christina Ross, Matvey Tsivian, Stephen J. Walker

**Affiliations:** 1 Atrium Health Wake Forest Baptist Medical Center, Winston Salem, NC 27101, USA; wwhitman@wakehealth.edu (W.W.); rbissette@vt.edu (R.B.); swalker@wakehealth.edu (S.J.W.); 2 Virginia Tech Carilion School of Medicine, Roanoke, VA 24016, USA; 3 Wake Forest Institute for Regenerative Medicine, Winston Salem, NC 27101, USA; chrross@wakehealth.edu; 4 Department of Urology, Medical University of South Carolina Medical Center, Charleston, SC 29425, USA; tsivian@musc.edu

**Keywords:** pulsed electromagnetic field therapy, bladder cancer, urology, therapeutic, HT-1197, non-invasive

## Abstract

**Background/Objectives:** Pulsed electromagnetic field (PEMF) therapy involves the use of magnetic waveform energy for targeted treatment delivery. This technique has shown promising results in the treatment of various cancers. Currently, treatment of bladder cancer is highly invasive, involving intravesical chemotherapy or radical cystectomy. The potential therapeutic effects of PEMF therapy on bladder cancer are a relatively new and understudied area; therefore, the goal of this investigation was to gain mechanistic insight by examining the effects of PEMF therapy on a bladder cancer cell line in vitro. **Methods:** Cells from the bladder cancer cell line HT-1197 were cultured and incubated with (treatment group) or without (control group) PEMF therapy for one hour each day for five days. Cell counts were compared using Incucyte^®^ data to determine proliferation rates. At days 1 and 5, total RNA was isolated from cells, and following quantity and quality checks, gene expression was compared between the two groups. Proliferation rates from cell line HT-1197 were compared to prior published results on the bladder cancer cell line HT-1376. **Results:** HT-1197 cells treated with PEMF therapy had slower proliferation rates compared to controls (*p* < 0.05), but HT-1376 cells did not (*p* > 0.05). Principal component analysis showed complete separation of treated and untreated cells, with PEMF treatment accounting for 76% of the variation between the groups. Expression of numerous genes and cancer-related pathways was altered in the treated cells relative to the controls. **Conclusions:** Bladder cancer HT-1197 cells treated with PEMF therapy had slower proliferation and corresponding changes in gene expression. Several cancer-relevant pathways were differentially regulated following PEMF treatment. The conclusions are limited by the lack of a control healthy urothelial cell line in the experiments. Despite this shortcoming, our results suggest that PEMF therapy may be a promising avenue for further research in the treatment of bladder cancer.

## 1. Introduction

Pulsed electromagnetic field (PEMF) therapy is a magnetic waveform energy that can be targeted for delivery to cells and/or patients [1,2]. PEMF therapy uses repetition frequency to activate a magnetic field, which then is activated for short intervals to treat cells or tissues [2]. While the exact mechanism(s) that underlies the ability of PEMF therapy to alter cells and tissues is yet to be fully explained, some theorize that a force is created on cells/tissues, which is related to the cell/tissue magnetic reactive properties and by making an electric field, resultsin the movement of charged molecules like calcium, potassium, and sodium [2,3]. The technology used to deliver PEMF therapy is variable across the literature, but for cellular studies, it most commonly involves the use of a Helmholtz coil, which creates a magnetic field that delivers a PEMF to the cells being studied [2]. There are numerous studies that describe the therapeutic effects of PEMF therapy on pain disorders and bone healing, both in vitro and in vivo [4,5,6]. More recently, studies exploring the use of PEMF therapy to treat various cancers have reported promising results [7]. Nevertheless, an understudied cancer with a high morbidity and mortality that still requires more research is bladder cancer.

Bladder cancer accounts for 3% of all globally diagnosed cancers each year, and in the United States is the sixth-most incident cancer [8]. Prognosis varies, with a 5-year survival of ~77%; however, the survival rate goes down to 5% for patients with metastatic disease [8]. Treatment options are invasive, ranging from intravesical therapies like Bacillus Calmette–Guérin or chemotherapy to major surgery like cystectomy for removal of the bladder. There is a strong need, then, for non-invasive therapies to treat patients afflicted with bladder cancer. Further, there is a paucity of published work examining how PEMF therapy effects bladder cancer cells. In the past, our research group has published on the effect of PEMF therapy on the bladder cancer cell line HT-1376, demonstrating a favorable change to the genomic profile of the PEMF-treated bladder cancer cells relative to those untreated [9]. Barbault et al. looked at a variety of different cancer cell lines, finding tumor-specific frequencies for optimal PEMF treatment, one of which was bladder cancer [10]. Aside from this, though, there are few reports of PEMF being used to treat bladder cancer.

The purpose of this study was to examine the effect of PEMF therapy on a bladder cancer cell line in vitro on both its genomic profile and proliferation rate.

## 2. Materials and Methods

### 2.1. Experimental Justification and Setup

**Figure 1:**
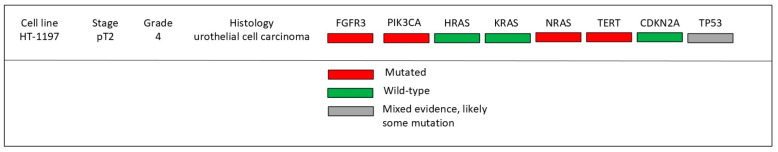
Mutations of bladder cancer cell line HT-1197. Tumor stage, grade, and histology are shown. Additionally, relevant genes that are either wild type or mutated are reported, with a legend corresponding to each gene. Figure adapted from Rasheed et al. [11].

The bladder cancer cell line HT-1197, a commercially available human-derived bladder cancer cell line from a patient who had urothelial carcinoma, was selected for this study [11]. HT-1197 cells have faster growth characteristics, higher grade, more oncogenic mutations, and more invasiveness than many other bladder cancer cell lines available for study (Figure 1) [11]. Mutations have also been characterized in this cell line and are available for review online [12]. The decision to use this cell line was made specifically with the hypothesis that if there is a benefit to be seen, then more aggressive dividing bladder cancer cells may be most susceptible to PEMF therapy and thus would be most likely to detect an effect. As noted, in the past, our research group has published on the effect of PEMF therapy on the bladder cancer cell line HT-1376, which is another urothelial cell carcinoma, but lower grade than HT-1197 and deemed to be more susceptible to chemotherapeutics like cisplatin [9,11,13]. The proliferation rates of HT-1376 PEMF and control cells, which have not been previously reported on, were included in this analysis for comparison with HT-1197.

### 2.2. Cell Culture

Cells were cultured according to standard protocols from the distributor website [14]. For HT-1197, Eagle’s minimum essential medium was used with fetal bovine serum for a final concentration of 10% to culture cells. Cells were incubated at 37 degrees Celsius with a 5% CO_2_ atmosphere for one week prior to conducting the experiment in order to obtain a sufficient number of cells for the remainder of the experiment. All cells utilized were passage 1 cells. There were two separate groups of HT-1197 cells utilized for this experiment, untreated cells (control group) and PEMF-exposed cells (treatment group). HT-1197 cells (passage 1) were seeded into two separate 12-well plates at a concentration of 27 K cells per well. The experiment was run in triplicates.

### 2.3. PEMF Delivery

**Figure 2:**
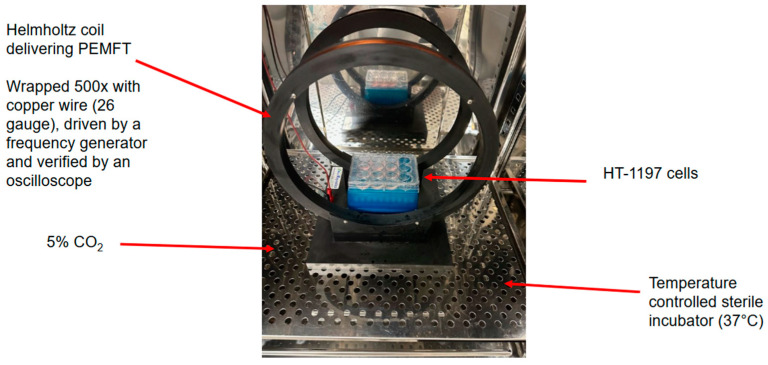
Experimental setup for pulsed electromagnetic field therapy administration. A Helmholtz coil was placed inside a temperature-controlled incubator. During treatment sessions, the culture plates containing the HT-1197 cells were placed at the same locale within the magnetic field, which was verified with a gauss meter. The field of treatment was determined to be uniform over the entire area of the culture plates.

All incubation of culture plates was performed in an Incucyte^®^ S3 Live-Cell Analysis System, which is an automated cell counter within an incubation system. Cell count analyses were performed at four-hour intervals for the duration of the experiment to determine proliferation rate and growth curves. One plate of the HT-1197 cells was removed from the Incucyte^®^ system and immediately placed into the PEMF treatment incubator. The incubator was equipped with a warm bath heated with a ceramic heating element to maintain a constant 37 degrees Celsius temperature. For delivery of PEMF therapy, an 11-inch diameter Helmholtz coil designed with 500 turns of 26-gauge copper wiring was used, which was connected to a frequency generator. The Helmholtz coil had an oscillating magnetic field ranging from 1.5 MilliTesla (mT) to 16 mT at 30 Hertz (Hz), and was used one hour each day for five total days in the incubator (Figure 2). An oscilloscope was used to measure frequency generated from the generator, and the range of intensities mentioned were the peak values within the treated area. Justification of magnetic field strength was from prior evidence published showing beneficial effects of PEMFs on multiple cancer cell lines using a magnetic field range like what was used in this analysis [15]. We elected to use a frequency of 30 Hz, which is classified as an extremely low-frequency PEMF [2]. This decision was made based on a prior study by Barbault et al., where the authors estimated the tumor-specific frequency of bladder cancer to be ~25 Hz and a paper by García-Minguillán and Maestú, where the authors concluded that a specific PEMF frequency of 30 Hz resulted in a reduction in viability of tumor glioma cells [10,16]. Once the one-hour treatment was completed, the treatment plate was immediately placed back into the Incucyte^®^ system to remain until the next treatment. Each treatment occurred exactly 24 hours after the previous one. A gaussmeter was used to test the magnetic field within the treatment incubator before applying PEMF treatment each day to ensure the efficacy of the magnetic field. The magnetic field was specifically measured in the center of the Helmholtz coil as well as at the level of the cellular plate and at the top of the coil and was noted to be uniform in its strength throughout the incubator (Figure 2). Cells were placed in the incubator to receive treatment in the sterile culture dishes. Control cell lines were also placed into the incubator for one hour each day for a total of five days, but the Helmholtz coil magnetic field was not activated.

### 2.4. Growth Curve Determination

Using Incucyte^®^ data, cell counts of control and treatment groups were plotted as a function of time to develop growth curves. The following equation was used to calculate exponential growth rate: Pe^rt^ = A. P is the initial starting cell count, A is the final cell count, r is the growth rate, e is Euler’s number(2.718281828459), and t is the time in hours. The curve was fitted using an exponential regression model and SPSS Statistics version 29 (Armonk, NY, USA). An independent-sample t-test was run to compare proliferation (growth) rate between control and experimental samples, also with SPSS Statistics. This same process was repeated for prior experimental data on the bladder cancer cell line HT-1376 to assess differences in proliferation rate by cell line aggressiveness.

### 2.5. RNA Extraction and nCounter^®^ Tumor Signaling 360™ Analysis

Ribonucleic acid (RNA) was extracted from cellular samples using methodology we have previously reported [17]. RNeasy^®^ MinElute™ Plus columns (Qiagen^®^) were used for RNA recovery from cell samples according to the manufacturer’s instructions. RNA was eluted from the columns in nuclease-free water. RNA quantity and purity were determined using a NanoDrop™ ND-1000 spectrophotometer by measuring absorbance at 260/280 nm, with a mean ± SD of all samples of 2.09 ± 0.02. RNA quality was determined using a bioanalyzer (Agilent Technologies, Palo Alto, California), with a mean RNA integrity number of 9.4 ± 0.24 for all samples. Labeled complementary deoxyribonucleic acid (DNA) generated from RNA was assayed on an nCounter^®^ Tumor Signaling 360™ panel.

To evaluate differential gene expression between treated (day 5 of experiment) and control cells (day 5 of experiment) from the nCounter^®^ Tumor Signaling 360™ panel data, we conducted principal component analysis (PCA) and hierarchical clustering using Qlucore Omics Explorer. Differential gene expression data were imported into Qiagen^®^ ingenuity pathway analysis (IPA) for identification of canonical pathways that were overrepresented in the dataset.

## 3. Results

**Figure 3:**
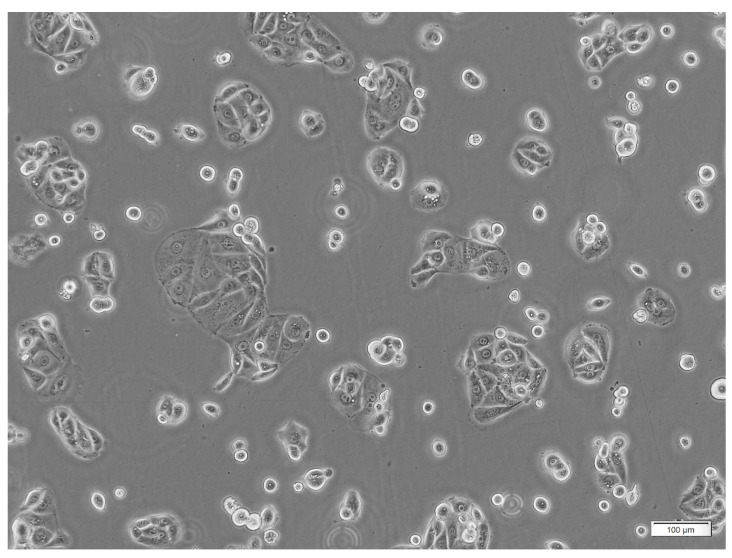
HT-1197 cells in culture. HT-1197 bladder cancer cells prior to beginning the experiment under a microscope at 10x magnification. Cells can be seen in clusters intact growing appropriately before the experiment was started. Both PEMF and control cells came from this cell line. A scale is provided in the bottom-right corner of the figure.

**Figure 4:**
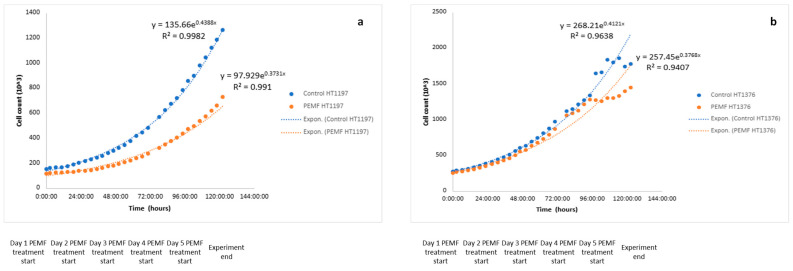
Cell growth curves. Representation of the exponential growth curves of both the control (blue) and PEMF-treated cells (orange). The x-axis is time from the start of the experiment in hours and cell count is on the y-axis. The 0:00:00 time point represents the start of PEMF treatment on day one of the experiment. On both curves, “e” represents Euler’s number, a constant value of 2.718281828459. (**a**) Exponential growth lines with equations are provided for HT-1197 PEMF and control cells. (**b**) Exponential growth lines with equations are provided for HT-1376 PEMF and control cells.

**Figure 5:**
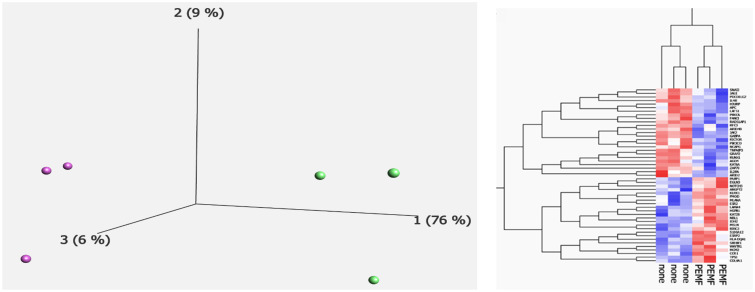
Principal component analysis and hierarchical clustering. (**a**) PCA was performed using gene expression profiles from control (purple) and experimental (green) samples. (**b**) Hierarchical clustering revealed 49 DETs between the groups. “None” represents the control cells and “PEMF” the experimentally treated cells. Red represents upregulated DETs (PEMF vs. control) and blue downregulated.

HT-1197 cells in culture from day 1 of the experiment prior to any PEMF treatment are presented in Figure 3. The growth curves for HT-1197 cells are shown in Figure 4a. R^2^ values for the PEMF and control cells were 0.99 and 0.99, respectively. PEMF-treated cultures showed a significantly slower proliferation rate compared to controls (Figure 4a, *p* < 0.05, (CI 4.8–27.5)). The growth rate of the treatment group was 0.0152 compared to 0.0176 of the control group. Figure 4b shows the growth curves for PEMF and control HT-1376 cells from prior experimentation [9]. R^2^ values for the PEMF and control cells were 0.96 and 0.94, respectively. No significant difference in growth rate was noted (Figure 4b, *p* = 0.353, (CI −108.7–39.4)). PCA revealed a complete separation between the two groups (Figure 5a), with PEMF treatment (PC1) accounting for 76% of the variation between the groups. There were 49 differentially expressed transcripts (DETs) found between control and PEMF-treated cells (Figure 5b). The heatmap reveals up- and downregulation of these transcripts in the PEMF-treated cells.

**Figure 6:**
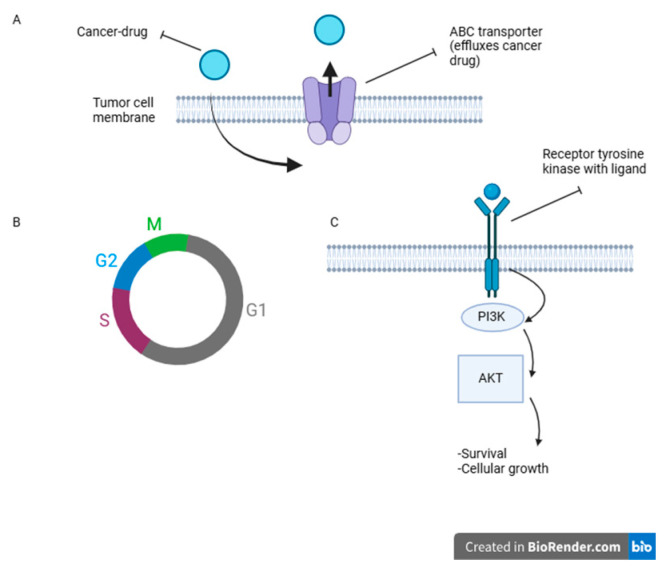
Cancer-relevant pathways. (**A**) The cancer drug resistance efflux pathway was downregulated in PEMF-treated cells. It normally works to remove the concentration of cancer drug inside a tumor cell. (**B**) The G1/S checkpoint regulation pathway, which acts to halt the cell cycle when damage to DNA is detected, was upregulated in PEMF-treated cells. (**C**) The PI3K/AKT pathway normally acts to promote cell survival and proliferation when activated. It was downregulated in PEMF-treated cells. Figure created with BioRender.com.

Data from IPA also revealed that many cancer-related pathways were altered after treatment with PEMF. Using gene ontology analysis, relevant cancer pathways downregulated after PEMF treatment in HT-1197 cells relative to controls were the tumor microenvironment (TME) pathway (*p* = 1.49 × 10^−41^), phosphatidylinositol 3-kinase (PI3K)/AKT pathway (*p* = 8.63 × 10^−43^), and cancer drug resistance efflux pathway (*p* = 4.5 × 10^−29^). Upregulated pathways after PEMF treatment relative to controls were the p53 signaling pathway (7.53 × 10^−52^), phosphatase and tensin homologue (PTEN) signaling pathway (*p* = 4.62 × 10^−40^), and the G1/S checkpoint regulation pathway (*p* = 2.3 × 10^−25^). Key cancer pathways are represented in Figure 6. Various cancer-relevant genes were also examined in IPA, showing differences in expression between control and PEMF-treated cells, with the logarithm fold change reported in Table 1. Key genes downregulated in PEMF-treated cells were JAK2, TGFβ2, and PIK3CD. Genes upregulated in PEMF-treated cells were Rb1, CDKN1A, and TP53.

## 4. Discussion

To our knowledge, this experiment represents the most comprehensive study to report on the effects of PEMF treatment on bladder cancer cells in culture. Our data show that PEMF treatment slowed the proliferation of the HT-1197 cancer cells relative to untreated HT-1197 cells. Moreover, PEMF treatment did not appear to have a significant effect on proliferation rate of a less aggressive bladder cancer cell line previously reported on, HT-1376. This seems to affirm our hypothesis that PEMF treatment’s effect is best in more aggressively dividing cancer cells. We demonstrated this graphically by two curves, with the PEMF-treated HT-1197 cells curve having a slowed maximal proliferation rate relative to their controls, but not for HT-1376. Nevertheless, based on prior data, PEMF treatment may still have a favorable effect on the genomic profile of less aggressive bladder cancer cell lines [9]. Using a similar experimental design, Pantelis et al. used breast cancer cells along with normal fibroblasts and administered PEMF treatment twice daily for 30 min each time, finding that PEMF treatment lowered cellular proliferation and viability in the cancer cells relative to untreated cells and the fibroblasts [18]. Furthermore, cellular senescence was induced in PEMF-treated breast cancer cells, but not controls or fibroblast cells [18]. Crocetti et al. also used PEMF treatment to treat breast cancer cells at different PEMF exposure intervals, noting cell damage to the cancer cells accrued with time and was significant after three days of treatment relative to control cancer cells, with an optimal duration of 60 min of PEMF treatment per day, which was our justification for treatment exposure times in our study [19]. Similarly, Akbarnejad et al. investigated the effect of PEMF treatment in glioblastoma multiforme cells in vitro, finding decreased proliferation and increased apoptosis in the PEMF-treated cells compared to controls [20]. Similarly to prior experiments in other cancer cell lines, we noted decreased cellular proliferation and slower growth in the PEMF-treated aggressive bladder cancer cells.

Differential expression in cancer-relevant pathways and genes were identified in this study. PEMF-treated cells demonstrated decreased expression of the TME, PI3K/AKT, and cancer drug resistance efflux pathways. The TME pathway is known to play critical roles in cancer initiation, progression, and eventual metastasis [21]. While data are limited, other studies similarly reported PEMF treatment to alter critical aspects of the TME, such as decreasing the angiogenesis ability of tumor cells and decreasing secretion of proinflammatory cytokines [22,23]. PI3K/AKT pathway activation has a well-established role in bladder cancer carcinogenesis [24] and it has been targeted for potential therapeutic interventions with promising results [25,26]. Our results indicate PEMF treatment downregulates this pathway and may have a future role in treating bladder cancer. An additional pathway, the cancer drug resistance efflux pathway, also had lower expression in PEMF-treated cells. In line with our findings, Xu et al. examined how magnetic field therapy affects chemotherapy in cancer cells, albeit not bladder cancer, noting that magnetic field treatment increased chemotherapy drug intake into cells and decreased drug efflux by inhibiting cell surface efflux transporters [27]. Bladder cancer has a high recurrence rate, and drug efflux mechanisms are felt to be critical culprits in this [28].

Upregulated pathways after PEMF treatment relative to controls were the p53 signaling pathway, G1/S checkpoint regulation pathway, and the PTEN signaling pathway. Woo et al. examined PEMF treatment’s effect on breast cancer cells in vitro, finding that PEMF activated p53, limiting cancer cell proliferation, which is a well-known tumor suppressor protein that inhibits the cell cycle and can lead to apoptosis [29]. The G1/S pathway also arrests the cell cycle when DNA acid damage is detected, and acts to prevent the accumulation of mutations in DNA by preventing cell cycle progression [30]. Alterations in this pathway have been observed in the majority of bladder cancers [30]. Interestingly, Woo et al. showed that PEMF treatment increased the chemotherapeutic drug doxorubicin’s effect on breast cancer G1-mediated cell cycle arrest and increased expression of G1/S pathway-related proteins like p53 [29]. Puzio-Kuter et al. demonstrated that PTEN inactivation promotes tumor invasion in bladder cancer in mouse models and correlates with poor survival outcomes in human bladder cancer patients [31]. Our findings similarly show PTEN upregulation in response to PEMF treatment [31].

Multiple cancer-relevant genes had expression changes in our IPA after PEMF treatment relative to controls. In PEMF-treated cells, JAK2, TGFβ2, and PI3KCD were downregulated. Others have also shown PEMF treatment to have similar effects on JAK2, leading to lower tumor-signaling inflammation [32]. TGFβ2 has shown mixed expression changes after PEMF treatment in cells, but its promotion is well implicated in higher bladder cancer stages, grades, and invasion potential [33,34,35]. Moreover, as discussed earlier, the PI3K/AKT pathway was downregulated in PEMF-treated cells, and so unsurprisingly a key gene target in that pathway (PI3KCD) was also downregulated. Genes upregulated in PEMF-treated cells were Rb1, CDKN1A, and TP53. Rb1 knockout mutations are well studied in bladder cancer, and have been linked to decreased apoptosis, higher T-cell inflammatory responses, and predict worse overall survival in bladder cancer patients [36,37,38]. We have shown that PEMF upregulates Rb1 in HT-1376 bladder cancer cells too [9]. CDKN1A is a gene encoding key tumor suppressors p53 and p21. Mutations in this gene are rare in other common cancers, but frequently seen in bladder cancer, making this result particularly interesting. Lastly, as expected, TP53 was upregulated with PEMF treatment as it is encoded by CDKN1A. TP53 was also upregulated in PEMF-treated HT-1376 cells in the past [9]. Further, our finding is in line with the literature, as prior PEMF experimentation has shown that treatment activates this gene [29].

There are several limitations worth acknowledging in our study. First, we recognize that this experiment was conducted in vitro. The results may not easily translate in vivo and require prospective treatment confirmation on bladder cancer patients. Second, not having healthy non-cancer urothelial cells included in the analysis limits the interpretation of our results and is required for additional investigation moving forward. Additionally, we acknowledge that using a single set of PEMF delivery parameters limits the generalizability of the findings. Our magnetic field strength decision came from prior literature to ensure our range was within established parameters of what has been shown to provide benefit to other tumor cells [15]. We did not attempt to increase the magnetic field in this study, but Crocetti et al. showed that continued increase in magnetic field strength did not provide anti-carcinogenic benefits, and there is likely an optimal magnetic field range for PEMF treatment to work within [19]. Some have also posited that low-frequency magnetic fields could be carcinogenic, but it is well established that other tissue lines like breast cancer cells show favorable responses to PEMF treatment in frequency levels even lower than that used in our study [18,39]. Nevertheless, we would benefit from analyzing a higher PEMF on the same cell line in our analysis. We would also benefit in the future by validating the gene expression changes using quantitative polymerase chain reaction to confirm the nCounter^®^ Tumor Signaling 360™ panel data. Lastly, while we demonstrate that PEMF caused genetic differentiation of HT-1197 and specific pathways that were affected, and the potential mechanism behind this requires further investigation.

## 5. Conclusions

To the best of our knowledge this is the first study to investigate PEMF effects on HT-1197 bladder cancer cells in vitro. We found that HT-1197 bladder cancer cells treated with PEMF had slower proliferation, genomic separation, and changes in gene expression favoring an anti-cancer profile after treatment. There was not the same slowed cellular proliferation with HT-1376 cells that we saw with HT-1197 cells, though. Additionally, a multitude of cancer-relevant pathways were up- and downregulated after PEMF treatment. Our results represent an intriguing avenue for further research on this important cancer. Future studies should include additional bladder cancer cell lines as well as healthy human urothelial cells, and we recognize the significant limitations to our conclusions due to the lack of control cells. Despite these shortcomings, this study lays the groundwork for a multitude of projects in the field of bladder cancer dealing with PEMF treatment.

## Figures and Tables

**Table 1:** Gene expression changes. Key cancer-related genes whose expression changed in the PEMF treatment cell line relative to controls. Each gene is listed along with its typical role in cancer pathogenesis. The log_2_ fold change is the expression change in the PEMF cell line relative to controls. A negative value is decreased expression and positive value increased expression.

Gene	Role in Cancer	Log_2_ Fold Change
JAK2	Non-receptor tyrosine kinase: activating mutations implicated in cell proliferation	−0.34
TGFβ2	Signaling complex: promotility factor in bladder cancer cells	−0.31
PIK3CD	Oncogene: promotes growth factor-independent growth and increases cell invasion and metastasis	−0.22
Rb1	Tumor suppressor: halts cell cycle, loss of function leads to unregulated cell growth	0.13
CDKN1A	Cyclin-dependent kinase: target of p53 and activation halts the cell cycle	0.10
TP53	Tumor suppressor: loss of function promotes cell division	0.15

## Data Availability

Data from this experiment are available online via the Gene Expression Omnibus (GSE292216, GSM8853265, GSM8853266, GSM8853267, GSM8853268, GSM8853269, GSM8853270). Additional data are not publicly available, but are available upon reasonable request to the corresponding author.

## Abbreviations

The following abbreviations are used in this manuscript:

PEMF	pulsed electromagnetic field therapy
mT	MilliTesla
RNA	ribonucleic acid
DNA	deoxyribonucleic acid
Hz	hertz
IPA	ingenuity pathway analysis
PCA	principal component analysis
DETs	differentially expressed transcripts
TME	tumor microenvironment
PI3K	phosphatidylinositol 3-kinase
PTEN	phosphatase and tensin
